# Optical Computing: Status and Perspectives

**DOI:** 10.3390/nano12132171

**Published:** 2022-06-24

**Authors:** Nikolay L. Kazanskiy, Muhammad A. Butt, Svetlana N. Khonina

**Affiliations:** 1 IPSI RAS-Branch of the FSRC “Crystallography and Photonics” RAS, 443001 Samara, Russia; kazanskiy@ipsiras.ru (N.L.K.); khonina@ipsiras.ru (S.N.K.); 2 Samara National Research University, 443086 Samara, Russia; 3 Institute of Microelectronics and Optoelectronics, Warsaw University of Technology, Koszykowa 75, 00-662 Warszawa, Poland

**Keywords:** optical computing, logic gates, neural networks, plasmonic switches, digital optical computing, analog optical computing

## Abstract

For many years, optics has been employed in computing, although the major focus has been and remains to be on connecting parts of computers, for communications, or more fundamentally in systems that have some optical function or element (optical pattern recognition, etc.). Optical digital computers are still evolving; however, a variety of components that can eventually lead to true optical computers, such as optical logic gates, optical switches, neural networks, and spatial light modulators have previously been developed and are discussed in this paper. High-performance off-the-shelf computers can accurately simulate and construct more complicated photonic devices and systems. These advancements have developed under unusual circumstances: photonics is an emerging tool for the next generation of computing hardware, while recent advances in digital computers have empowered the design, modeling, and creation of a new class of photonic devices and systems with unparalleled challenges. Thus, the review of the status and perspectives shows that optical technology offers incredible developments in computational efficiency; however, only separately implemented optical operations are known so far, and the launch of the world’s first commercial optical processing system was only recently announced. Most likely, the optical computer has not been put into mass production because there are still no good solutions for optical transistors, optical memory, and much more that acceptance to break the huge inertia of many proven technologies in electronics.

## 1. Introduction

The topic of optical computing (OC) dates to the early 1960s, or perhaps before when the military became interested in utilizing the Fourier transform (FT) relationships essential in coherent optical imaging systems to perform processes such as convolution and correlation. On data supplied in imaging the optical format to a bulk optical system, it was easily demonstrated that these processes could be performed with considerable speed. Such processors were fundamentally analog in operation, and as a result, they constantly struggled to maintain adequate dynamic range and signal-to-noise ratios, severely restricting their use. Despite several spectacular demonstrations, silicon digital electronic processing appears to have nearly always been chosen for final manufacturing equipment [1]. Nonetheless, there is still a lot of interest in such specialized machines, and it is certainly true that they can attain very high comparable digital processing speeds. The use of nonlinear optical tools to establish the basic digital processing functions of AND, OR, NAND, NOR, etc., sparked even more interest in the early 1980s, this time in imaging optical format, where a single lens could, in theory, image a very large number of parallel channels from a 2D array of devices. As a result, assertions have been made that those future high-speed computers would use vastly parallel digital–optical processing to attain speeds much above those conceivable with electronics [2].

Following that, a slew of large R&D initiatives aimed at capitalizing on the potential. Such statements are established on basic expectations about prospective digital throughput, but they easily disregard the enormous practical issues that come with putting them into practice. Because the analog light wave level represents the digital state, practically all-optical logic systems addressed in the literature use threshold logic, meaning highly tight control of the optical power level across a complicated multichannel system. Given how readily unanticipated 3 dB insertion losses may be obtained in complicated systems of mirrors, holograms, lenses, and other components, one would wonder if this had any chance of being realized. Nevertheless, employing a dual-rail optical signaling system, the symmetric self-electrooptic effect device (SEED) technique established at AT&T Bell Labs and explored elsewhere does give a sophisticated resolution to this problem [3]. Other important concerns include the fact that the finest optical logic systems are optically activated electrical equipment, and huge ones at that because light must enter them, and optical wavelengths are extremely long by electronic norms. The practical concerns of compiling high-resolution imaging optical systems, the shallow depth of field, the accuracy inferred in the lenses (in terms of focal length), and the mechanical tolerances and massive dimensions of constructed structures are all simple issues that are conveniently overlooked by many. Nevertheless, none of these disadvantages indicates that it cannot be achieved; rather, they raise the challenge, and as some of the results will demonstrate, genuinely spectacular experimental procedures established on free-space optics may be created in the research laboratory.

The switches, optical logic gates (OLGs), and memory components that regulate the flow of electrons in an electronic computer would be replaced by optical systems in a digital optical computer. Optical modulators, which come in a variety of shapes and sizes, may perform the roles of the OLGs and switches. A 1 × 1 switch is a simple on–off switch with the ability to link two lines. A 1 × 2 switch connects one line to one of two lines. Two lines are connected by a 2 × 2 switch. It can be in one of two states: input (I/P) 1 linked to output (O/P) 1 and I/P 2 connected to O/P 2, or I/P 1 connected to O/P 2 and I/P 2 connected to O/P 1, which is known as the bar state, or I/P 1 connected to O/P 2 and I/P 2 connected to O/P 1, which is known as the cross-state. As a result, it is known as a crossbar switch. A crossbar switch may be stretched to *n* × *n* arrangement, which allows any of *n* I/P lines to be linked to any of *n* O/P lines at any time without causing interference.

Optical switches can be made from electro-optic modulators, acousto-optic modulators, magneto-optic modulators, and liquid crystals, among other forms of optical modulators. A crossbar switch can also be made from an array of modulators. An array of *n* × *n* light valves, for instance, might be used to create an *n* × *n* crossbar switch. The light source would be a vertical linear array of *n* laser diodes. The light from the diodes is dispersed horizontally by a cylindrical lens such that each diode illumines one row of the *n* × *n* array. Then, one lens per row of the modulator array, a set of *n* cylindrical lenses, positioned perpendicular to the first lens, is employed to direct the signal emitted by the array onto a horizontally oriented linear array of *n* photodetectors. This setup allows light from any laser diode to be connected to any detector without interruption. Switches that are operated both electrically and optically have been designed. Optically regulated apparatuses would be used in an all-optical computer. The switches should also be tiny, high-speed apparatuses that can be produced in vast arrays and consume very little switching energy. Several alternative technologies have been used to illustrate the needed switching functionalities. However, massive, high-density arrays of OLGs are currently being developed.

A multinational research team led by Russia’s Skolkovo Institute of Science and Technology has developed an exceptionally energy-efficient optical switch in conjunction with IBM [4]. The switch is extremely rapid and does not involve any cooling. It might serve as the foundation for the next generation of computers that manage photons instead of electrons. A 35-nanometer semi-conducting polymer consisting of organic material is placed between highly reflecting surfaces to form the switch. This results in the formation of a tiny chamber that traps light beams. The gadget is powered by two lasers: a pump laser and a seed laser. When the pump laser shines on the switch, thousands of indistinguishable quasiparticles develop in the same area, generating a Bose–Einstein condensate—a collection of particles that individually act like a single atom. The seed beam is utilized to alter this condensate between two measured states that act as binary codes ‘0′ and ‘1′. The new technology can do 1 trillion calculations per second, which is 1000 times quicker than today’s finest commercial transistors. Furthermore, it requires significantly less energy to transition states than transistors. This is due to the optical switch’s ability to be actuated by a single photon of light. Equivalent electrical transistors that employ single electrons typically need enormous quantities of a cooling device that uses a lot of electricity. The new switch, on the other hand, will function at room temperature. Before it can be deployed, the technology has a long way to go. The first electronic transistor takes years to make its way into a personal computer. The researchers face a hurdle in that, despite requiring relatively little energy to switch, the device still requires continual input from the pump laser. The team is researching ways to get around this by employing superfluorescent perovskite supercrystals to aid reduce power usage. Despite the difficulties, the researchers expect that the novel switch will be employed in various types of optical computing systems in the near future, maybe as a method to ramp up supercomputer processing [4].

The urgent demand for optical technology relies on the fact that the time response of electrical circuits limits today’s computers [5]. A solid transmission medium restricts signal speed and volume while also generating heat that destroys components. A one-foot length of wire, for instance, creates roughly one nanosecond (billionth of a second) of time delay. The extreme downsizing of microscopic electronic apparatuses also causes ‘cross-talk’ or signal mistakes that compromise the system’s dependability. These and other challenges have prompted researchers to look for solutions in light itself. Light does not even have the time domain restrictions of electronics, does not require insulators, and may even deliver dozens or hundreds of photon communication streams at distinct color frequencies at the same time. Those that are not susceptible to EM interference do not experience electrical short circuits. They feature low-loss transmission and a broad bandwidth; that is, they can communicate with numerous channels simultaneously without interference. They may transmit signals inside the same or neighboring optical fibers (OFs) with little to no interference or cross-talk. They are smaller, lighter, and less costly to produce, and they work better with stored data than magnetic materials. Scientists want to construct a new class of computers that run 100 million times quicker than today’s apparatuses by restoring electrons and cables with photons, OF, crystals, thin films, and mirrors [6].

Optical processing hits at a critical juncture in computing history. The need for artificial intelligence (AI) is growing at the same time as Moore’s Law is breaking down in silicon-based computing. Optalysys is a game-changing technology startup that uses groundbreaking optical processing techniques to address this challenge, allowing new levels of AI capability for high-resolution image and video-based applications [7]. The technology is emerging to speed up several of the most demanding processor-intensive operations while using a fraction of the energy that silicon processors consume. It may be configured to execute optical correlation and convolution operations using the application programming interface (API) or Tensorflow interface to unleash new levels of AI and pattern recognition potential. Optalysys employs photons instead of electrons, but it also uses high-resolution computations, allowing huge image/pattern-based data to be handled at speeds significantly quicker than silicon. The Optalysys technology can deliver something distinctive in the API space: a scalable processor that can accomplish end-to-end, full-resolution processing of multi-megapixel image and video data, or contextually pre-process data for enhancing the effectiveness of existing Convolutional Neural Network (CNN)-type models for high-resolution data applications, thanks to the characteristics of diffractive optics.

## 2. Types of Computing and Necessity of Optics for Computing 

OC may be divided into two types: digital optical computing (DOC) and analog optical computing (AOC). For more than 30 years, DOC established on Boolean logics has been established, employing a technique comparable to general-purpose computing established on transistors. Nonetheless, given reduced optical device integration density, it is impossible to beat traditional digital computing. AOC, on the other hand, makes use of the physical characteristics of light, including amplitude and phase, as well as the interactions between light and optical apparatuses, to perform specific computations. Due to the obvious unique mathematical portrayal of computational processes in one AOC system, it is considered dedicated computing. AOC can achieve higher data processing speed in specialized applications, including pattern recognition and numerical calculation, as compared to standard digital computing. As a result, AOC systems have received a lot of academic attention as one of the most potential pervasive computing in the post-Moore age.

Optical technology offers tremendous improvements in computational efficiency and speed along with considerable size and cost reductions [8,9]. Since many processes may be conducted at the same time, an optical desktop computer might process data 100,000 times quicker than existing versions. Low production costs, resilience to electromagnetic (EM) interference, low-loss transmission tolerance, independence from short electrical connections, and the capacity to deliver wide bandwidth and transport signals without interference within the same or neighboring OFs are all features of optics. The distinction between optical and electrical synchrony may be shown with a simplistic example. Imagine an imaging system with 1000 × 1000 distinct units per mm^2^ in the object plane that are optically coupled to a comparable number of points per mm^2^ in the image plane; the lens essentially conducts a real-time fast fourier transform (FFT) of the image plane. A million processes are necessary to do this electronically. If parallelism is combined with rapid switching speeds, startling computing speeds are possible. Consider there are only 100 million OLGs on a chip, which is significantly fewer than the figure indicated above. Furthermore, imagine that each OLG has a switching period of just 1 nanosecond (organic optical switches may switch at sub-picosecond rates, but electronic switching has a maximum picosecond switching time). Well over 10^17^-bit operations per second may be performed by such a device. When compared to the gigabits (10^9^) or terabits (10^12^) per second speeds that electronics are now restricted to or aiming for, this is a significant difference. In other words, an operation that would take a normal computer a hundred thousand hours (>11 years) might just be completed in less than an hour by an optical computer.

As photons are uncharged and do not react with each other as easily as electrons, light does have another benefit. As a result, in full-duplex functioning, beams of light can travel across each other without altering the information transmitted. Loops in electronics produce noisy voltage spikes anytime the EM fields pass through the loop change. Furthermore, switching pulses with a high frequency or a short duration will generate crosstalk in surrounding lines. Signals in nearby OFs or optical integrated channels are unaffected by each other, and they do not pick up noise from loops. Ultimately, optical materials outperform magnetic materials in terms of storage density and availability. The subject of OC is growing quickly and offers many exciting possibilities for transcending the limits of today’s electrical computers. Optical apparatuses are already being integrated into a variety of computing systems. Resulting in mass manufacture, the cost of laser diodes as coherent light sources has reduced dramatically. Optical CD-ROM discs have also been widely used in both home and office PCs [10].

## 3. Optical Processing Architecture

Optical information processing is introduced and established by utilizing all of light’s attributes of speed and parallelism to handle data at a high rate. The data are represented as a light wave or graphic. The inherent parallel processing of OC was frequently noted as a fundamental benefit of optical processing over electronic processing employing predominantly serial processors. As a result, optics hold significant promise for interpreting enormous amounts of data in real time. OC is established on the FT feature of a lens. When employing coherent light, a lens executes the FT of a 2D transparency in its front focal plane in its rear focal plane. The lens computes the precise FT with amplitude and phase in an analog method [11].

The I/P plane, the processing plane, and the O/P plane are the three planes that make up the system. The I/P plane displays the data to be processed; this plane will usually implement an algorithm. Conversion from electricity to light is performed via a Spatial Light Modulator (SLM). A 1D or 2D signal can be used as an I/P. In the context of a 1D I/P signal, an acousto-optic cell is commonly utilized, whereas 2D SLMs are used for 2D signals. Due to the lack of SLMs in the early years, the I/P plane consisted of a stationary slide. As a result, the concepts and possibilities of optical processors could be proved, but no real-time applications could be presented, rendering the processor worthless for most real-world applications. Lenses, holograms (optically recorded or computer produced), and nonlinear components can all be found in the processing plane. This is perhaps the most important component of the processing, and it can be performed at the speed of light in most optical processors. The O/P plane, which detects the processing results, is made up of a photodetector, a photodetector array, or a camera. Since the bulk of them are running at video rates, the speed of the entire process is restricted by the speed of its slowest component, which is usually the I/P plane SLM. The SLM is an essential component in the creation of realistic optical processors, but it is also one of their weakest. Likewise, the poor performance and expensive price of SLMs have slowed the development of a real-time optical processor.

**Figure 1:**
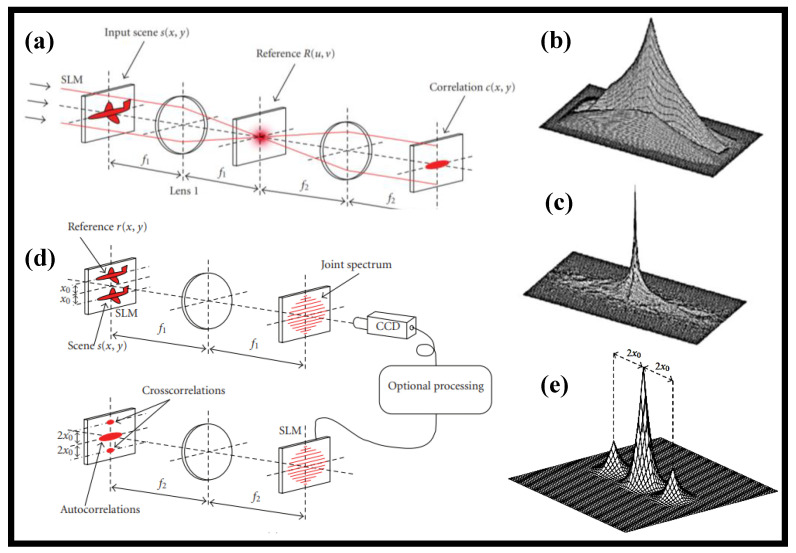
(**a**) Optical setup of basic 4-f correlator [22], (**b**) autocorrelation peak for a matched filter [22], (**c**) autocorrelation peak for a phase-only filter [22], (**d**) optical setup of JTC [22], (**e**) O/P plane of the JTC [22].

Real-time pattern recognition was first thought to be one of the most potential technologies of optical computers; hence, the following two optical correlator designs were developed. Because the separation between the I/P plane and the O/P plane is four times the focal length of the lenses, the basic correlator is termed 4-f in Figure 1a. This extremely simple design is established on Marechal and Croce’s spatial filtering work in 1953 [12] and was further refined by numerous researchers over the years [13,14]. The I/P scene is projected in the I/P plane, and Lens 1 performs the FT. The reference’s actual conjugated FT is placed in the Fourier plane and hence multiplied by the I/P scene’s FT. Lens 2 uses a second FT to determine the relationship between the I/P scene and the reference in the O/P plane. The fundamental issue of this arrangement was developing a sophisticated filter using the reference’s FT, and Vander Lugt suggested in 1964 to utilize a Fourier hologram of the benchmark as a filter [15]. Figure 1b,c depict the O/P correlation peak for autocorrelation when the correlation filter is a matched filter and when the correlation filter is phase-only, respectively.

Weaver and Goodman [16] proposed a new optical correlator layout in 1966, the joint transform correlator (JTC), which is shown in Figure 1d. The two pictures, the reference *r*(*x*,*y*) and the scene *s*(*x*,*y*), are arranged beside each other on the I/P plane, which is the first lens FTs. After detecting the intensity of the combined spectrum, the FT is used. The cross-correlations between the scene and the reference are among the terms that make up the second FT. This FT may be completed optically using an SLM, as seen in Figure 1d. When the reference and scene are similar, the O/P plane of the JTC is shown in Figure 1e. The two cross-correlation peaks are the only ones worth looking at. The CCD camera can be exchanged with an optical component such as an optically addressed SLM or a photorefractive crystal to create a completely optical processor. Because the JTC does not need the computation of a correlation filter, it is the appropriate design for real-time applications including target tracking where the benchmark must be revised at a rapid rate. Coherent optical processors are illustrated in Figure 1. Wave intensities, rather than complex wave amplitudes, are used to carry information in incoherent optical processors. Incoherent processors are not affected by phase changes in the I/P plane and do not produce coherent noise. The non-negative real value of the information, on the other hand, necessitates the employment of a variety of methods to execute some image processing applications [17,18]. Linear optical processing may be broken down into space-invariant activities such as correlation and convolution as well as space-variant operations such as coordinate transformations [19] and the Hough transform [20]. Logarithm transformation, thresholding and analog-to-digital conversion are examples of nonlinear processing that may be performed optically [21].

## 4. Types of Optical Components Used to Implement Optical Functions

Deep learning has an unquenchable thirst for processing resources. Classical deep learning has silently developed a bottleneck due to the interference of electrical impulses, energy usage, and physical constraints [23], even though electronic parts established on silicon can still sustain it presently. Academic and corporate circles are attempting to find new approaches for resolving electrical flaws that are less computationally intensive. It has huge benefits in information transmission and OC due to its high speed of 300,000 km per second, which is 300 times faster than that of an electron, and its data-carrying capacity and variety, which is 2 × 10^4^ times greater than that of electric channels, as well as high parallelism and strong anti-interference [24]. Switching electricity with light has emerged as a viable and sustainable work style, following the current trend.

### 4.1. Spatial Light Modulator (SLM)

Light wave processing has provided useful techniques for converting data into spatially modulated coherent light waves using SLM apparatuses, allowing the creation of digital holographic images [11]. The capacity to alter the phase and amplitude of light in the far-field is one of the hologram’s most valuable qualities. The FT illustrates how a hologram (near field) interacts with its replay field (far-field). In free space, the far-field might develop at the focal point of a positive lens or an indefinite distance from the near field plane [25]. Waveforms from an existent item can be reproduced using holograms. With developments in digital technology and light wave processing, it is now feasible to compute interference patterns computationally and construct synthetic wavefronts of any shape. Computer generated hologram (CGH), diffractive optical elements (DOE), phase/amplitude masks, diffractive grating, and other terms can be used to describe these interference patterns. Because they all work on the concept of diffraction, the nomenclature is rather arbitrary.

**Figure 2:**
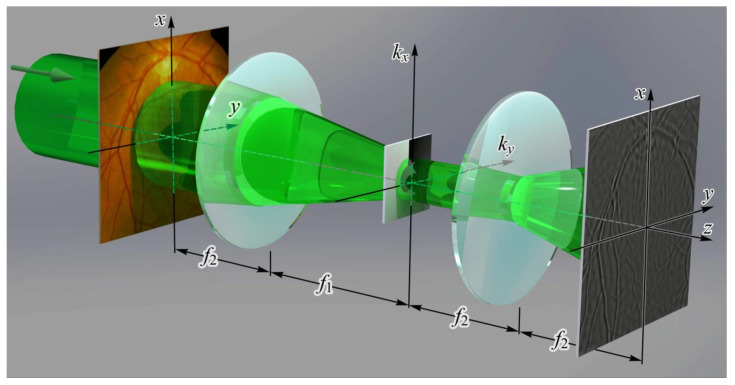
Illustration of the optical implementation of the radial Hilbert transform using the spiral phase plate as a spatial filter to contrast the image of the fundus vessels [51].

CGHs and DOEs have historically been used to implement various optical operations in correlators as spatial filters [26,27,28]. Despite the advent of dynamically controlled SLMs, the use of DOEs in Fourier correlators remains relevant in high-energy applications [29] since they have a significantly higher damage threshold than SLM. Soifer et al. have designed a multichannel spatial optical that allows coherent light fields to be optically decomposed into a series of orthogonal functions: using angular harmonics to calculate the light field’s angular momentum [30,31,32,33,34], detection and analysis of wavefront aberrations using Zernike polynomials [35,36,37,38,39], and using the optical Karhunen–Loeve decomposition, we may derive decorrelated image characteristics [40,41]. Relying on segmented spatial filters in the sequence of diffraction gratings, an optical approach for producing a directions field for fringed/contour pictures such as interferograms and fingerprints has been established [42,43,44,45]. To deepen focus and adjust for defocusing and chromatic aberrations, imaging systems undergo phase apodization and optical wavefront coding [46,47,48,49]. The spiral phase plate as a phase rotor filter is suggested to be used to visually realize the *m*-th order Hankel transform as well as to optically discriminate the light field with rotational symmetry [50]. In contrast to the commonly used 2D Hilbert transforms in image processing, the vortex spatial phase filter is utilized to execute the radial Hilbert transform, which is isotropic [51,52,53]. Figure 2 shows the operation of the optical correlator with a spatial filter in the form of the spiral phase plate for the implementation of the radial Hilbert transform to contrast the image of the fundus vessels [51]. In this way, a correlation filter can be used not only for the classical task of optical detection (recognition) (Figure 1) but also for the optical implementation of a mathematical operation such as Hilbert transform (Figure 2).

The SLM is an electrically programmable device that may modify light in compliance with a fixed spatial (pixel) pattern. It may typically be used to adjust the phase and/or amplitude of incoming light. As a result, SLM may easily realize phase-only, amplitude-only, or a combination of phase-amplitude [54]. Commercially available SLMs are quite “slow”, although faster ones are also known [55]. A DMD (digital micromirror device) is considered a fast analogue of SLM. DMDs show switching speed ranging from several kHz to tens of kHz (settling time for full-scale angle change is around 10 μs) [56]; however, high speed comes at the expense of limited modulation depth and diffraction efficiency [57]. Thus, in applications where high energy efficiency is not required, it is possible to use DMDs instead of SLMs. There are a variety of modulation techniques to choose from. EO-SLM is one of the most appealing and commonly utilized. Liquid crystal is used as the modulation material in EO-SLM. A micro-display is used for incident light manipulation and collecting in a liquid crystal SLM as well. This may be completed in two ways: as a transmissive display using LCD SLM technology or as a reflective display using LCoS SLM technology. The liquid crystal molecular alignment is one of the modulator’s most prominent characteristics. This might take the form of a vertical, parallel, or twisted structure. As a result, with the right polarizing optics, the incident beam of light parameters, such as amplitude, phase, or their combination, may be efficiently adjusted.

The nematic LCoS technology is a form of SLM that allows for phase-only modulation [58]. Furthermore, it belongs to the electrically addressed reflection modulator class, in which the liquid crystal is controlled by a direct and precise voltage, and the beam of the light wavefront may be adjusted as well. The LCoS SLM may be used to rebuild pictures from CGH as a diffractive modulator [59]. CGHs may be used for a variety of communication applications, and it is increasingly being used in indoor visible light communication networks [60]. Additionally, a range of optimization approaches, including the iterative Fourier transform algorithm (IFTA), linear Fourier transform (i.e., linear phase mask), simulated annealing, and the Gerchberg–Saxton algorithm, can be used to quickly construct acceptable holograms [61,62]. The beam of the light wavefront can be modified when the SLM is used as a diffractive device for reconstructing graphics from CGH.

As previously stated, LCoS displays have gained a lot of traction as potential microdisplays for a variety of SLM applications. Likewise, they have appealing and important characteristics such as excellent spatial resolution and light efficiency. As a result, they have been employed in a wide range of optical utilizations, including communication, reconfigurable interconnects [63], storage [64], diffractive optics [65], metrology [66], and quantum computing [67]. They can also be used for light wave processing and monitoring in wave shaper technology [68]. Another feature of the LCoS is that it is extremely cost-efficient and can be configured in a variety of ways. Several functionalities such as group delay ripple correction, wavelength filtering, and chromatic dispersion correction are made possible by this. In addition, LCoS technology may be used in flex grids, which has been regarded as a key component for next-generation networks [64]. The classic fixed grid with 50 GHz spacing, as regulated by the International Telecommunication Union (ITU) Telecommunication Standardization Sector (ITU-T), has a variety of drawbacks. The fixed grid has been reported to result in poor usage of the optical spectrum. Furthermore, it severely limits the system’s transmission capacity. The flex grid implementation, on the other hand, allows for the use of several modulation formats and their coexistence on a common infrastructure. They can also be multiplexed densely and effectively, which helps optical networks not only extend their reach but also increase the per-channel data rate. It is also expected that the deployment of WSS and SDM would considerably aid in the expansion of the network’s coverage and capacity [64].

Electrically addressed SLMs (EASLMs) and optically addressed SLMs (OASLMs) are two types of SLMs that differ in how data are loaded into the apparatuses [69]. The EA has been the most prominent approach in modern commercial SLMs because of the growth of electronic information technology over the last few decades. However, because data must be converted back and forth between the optical and electrical domains, EASLMs are not the greatest solution for future all-optical information processing systems. The OASLMs, on the other hand, enable light to be modulated directly by light without having to go through an electronic–optical transformation [70,71]. Additionally, OASLMs are required for a variety of all-optical purposes that EASLMs cannot handle, such as coherent to incoherent image conversion, real-time optical correlation, and parallel all-optical processing [8,72,73,74,75]. OASLMs may be built in theory using material nonlinearities, with modulation over the read light achieved by spatially precisely modifying the characteristics of the materials through nonlinear optical stimuli [76]. Natural materials, on the other hand, have insufficient nonlinearities to permit efficient “light-control-by-light” inside nanoscale volumes. This renders the apparatuses exceedingly bulky or necessitates a lot of pumping power to collect big enough nonlinear modulations, making them unsuitable for the nano-era.

The recent remarkable advancement in dynamic optical metasurface (MS) technology gives a chance to overcome the challenges and proposes a unique framework for nanoscaled SLM [77,78]. The MS can also enhance optical engagements by focusing light on nanoscale volumes, allowing for the greater control of light fields in response to external mechanical, chemical, and magnetic stimuli, for example. The ultracompact EASLMs established on MS have recently been observed relying on the active manipulation of beams of light by additional electrical fields [79]. An OASLM established on MS-OASLM is proposed in [80], with a nonlinear polarization manipulation of read light by another write light at the nanoscale as the operating mechanism. It delivers 500-line pairs/millimeter (equivalent to a pixel size of just 1 μm), which is more than 10 times greater than a standard commercial SLM. The MS-OASLM has exceptional compactness and a thickness of just 400 nm. MS-OASLMs such as this might pave the way for next-generation all-optical data processing and high-resolution display technologies.

### 4.2. Plasmonic Switches

Moore’s law is expected to surpass its physical constraints, putting modern computer systems centered on von Neumann architecture at a physical limit [81,82]. Potential alternate prospect next-generation computing approaches are necessary due to the rapidly rising development of bandwidth needs with lower power utilization. As a result of the usage of optical interconnects in high computing chips rather than electrical interlocks to minimize power consumption and increase speeds, photonic computing is expected to have a competent replacement for existing electronic computing systems. Furthermore, Si photonics has considered a leading field owing to its capacity to provide effective light modulation, light confinement, and compliance with today’s CMOS production techniques [83,84]. A decade ago, the shift from electronic to photonic systems began. Related to current material discoveries, fabrication technique advances, and more ongoing research efforts in this field, photonic interconnects and circuits have witnessed significant advancements in recent years.

Photonic logic circuits rely heavily on optical switching and modulation. Optical computations are established on photonic logic circuits. Utilizing the thermo-optic effect, free carrier dispersion effect, and Pockels electro-optic effect, intensive research in the direction of efficient optical switches, optical modulators, and optical logic circuits has been presented [85,86,87,88]. All these tools are volatile, requiring constant voltage to manage, and as a result, they consume a lot of power. Furthermore, due to their low electro-refractivity, they have long interaction durations. As a result, these apparatuses have substantial footprints, making ultra-compact designs difficult to achieve [89,90]. A novel type of material known as phase change materials (PCMs) has found considerable application in both the electronics and photonics domains in recent years [91,92]. With high-speed switching between two steady states, PCMs reveal changes in electrical and/or optical characteristics. Several photonic and plasmonic apparatuses have been proposed and investigated, including on-chip optical modulators and optical switches established on PCMs [93,94]. One of the widely used PCMs in photonic apparatuses is Ge_2_Sb_2_Te_5_ (GST) [95].

Even though effective energy non-volatile (NV) memory and OLGs based on PCMs have been thoroughly researched and established over the last 20 years, PCM-based photonic NV memories and photonic logic circuits have received little attention. Novel NV combinational and sequential logic circuit designs are investigated as well as NV hybrid electro-optic plasmonic circuits. The electro-optic devices are made up of a plasmonic waveguide (WG) with a mono PCM layer. Changing the phase of the PCM between amorphous to crystalline likewise changes the optical losses in the WG. Electrical threshold flipping or thermal conduction heating via externally applied radiators or the plasmonic WG metal itself as an integrated heater can be used to generate phase shift in the PCM. All OLGs, a half adder circuit, and sequential circuits may be built employing plasmonic switches as active components, as illustrated. Furthermore, the plasmonic switches and logic functions have minimal extinction ratios larger than 20 dB, are small, have little operational power, and operate at high speeds. To develop an effective architecture for logic processes, photonics, plasmonics, and electronics are merged on the same platform.

**Figure 3:**
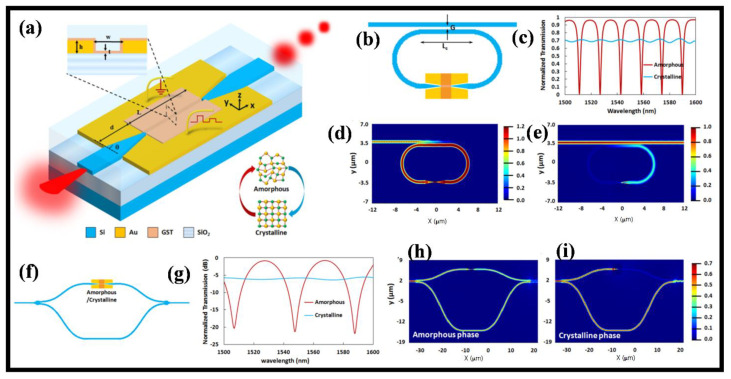
(**a**) The broadband NV hybrid EO plasmonic switch is shown schematically. The side view picture of the plasmonic slot WG with a thin coating of GST is shown in the inset [96], (**b**) The hybrid NV EO-plasmonic switch serves as the central unit in the racetrack μ-RR-based EO switch [96], (**c**) Normalized transmission spectra for the amorphous and crystalline phases of the PCM layer of the racetrack μ-RR-based EO switch, (**d**,**e**) The E-field mapping of the EO switch for the amorphous phase and the crystalline phase of the PCM layer at the resonance wavelength of 1558 nm, respectively [96], (**f**) Asymmetric MZI-based EO switch with hybrid NV EO plasmonic switch as an active component in one arm, (**g**) Normalized transmission band for the crystalline and amorphous phases of the PCM layer in the plasmonic switch for the asymmetric MZI based EO switch. The E-field distribution of the EO switch for the (**h**) amorphous phase and (**i**) crystalline phase of the PCM layer at the resonance wavelength of 1548 nm [96].

A plasmonic slot WG with PCM as an active material coating the slot WG’s surface is used in the NV hybrid electro-optic plasmonic switch [96]. Figure 3a depicts the suggested plasmonic slot WG’s construction, which includes Si-Au tapered WGs, a slot in an Au film to form a plasmonic slot WG, and a PCM coating for instance GST on the slot WG’s surface. The arrangement is made up of three primary components, the first of which is a dielectric to plasmonic mode converter in the I/P region of a hybrid Si-Au tapered WG. The major optical modulation happens in the second portion, which is an MIM plasmonic slot WG covered with a thin coating of GST, and the third part is a plasmonic to dielectric mode converter in the O/P section, which uses a hybrid Au–Si tapered WG. The cross-sectional image (in the y–z plane) of the GST-coated plasmonic slot WG is shown in the left top inset of Figure 3a [96].

As illustrated in Figure 3b, the EO switch is established on a racetrack micro-ring resonator (μ-RR) with an NV hybrid EO plasmonic switch in the ring WG as an active component. The optical mode is linked to the ring through a Si WG with the same cross-section as the hybrid plasmonic switch’s Si WG. The transmission spectra for both phases are shown in Figure 3c. The optical loss in the ring is minimal when GST is in the amorphous phase. Consequently, light can pass through the RR and propagate via the optical mode. For specific wavelengths, resonance is obtained in the RR, and after a roundtrip, light coupled back to the I/P WG’s throughport undergoes a 180° outphasing in comparison to light arriving from the I/P WG’s I/P point. As a result, destructive interference develops between light flowing from the I/P point and light coupled back to the I/P WGs through the port. As a result, light departing the O/P port at resonance wavelengths is eliminated, as shown in Figure 3d. The optical mode, on the other hand, cannot travel via the ring WG due to momentous optical loss in the crystalline phase. Consequently, there is no intrusion, and optical power is sent via the O/P port, as shown in Figure 3e [96].

Established on the hybrid plasmonic switch anticipated in [96], an asymmetric MZI is depicted in Figure 3f. The asymmetric MZIs were designed using compact and low-loss Y-junction-based splitters and combiners. The I/P segment’s splitter divides the I/P power evenly between the MZI’s two arms, while the O/P section’s combiner merges the optical powers of the two arms. The asymmetric MZI’s hybrid plasmonic switch is incorporated in the sorter arm. Figure 3g–i shows the transmission spectra and field mappings of the MZI for GST amorphous and crystalline phases, respectively.

### 4.3. Neural Networks (NNs)

Artificial intelligence, as among the most effective areas in computer science, focuses on simulating the framework of the nervous system by constructing artificial neural networks (ANNs), which maintain connections between neurons in multiple layers of the NN and give it higher accuracy and robustness. ANN’s research has advanced significantly since the 1980s. It has also effectively solved many functional challenges for modern computers in the fields of pattern recognition, intelligent robots, automatic control, prediction and estimation, biomedicine, economy, and other fields, all while retaining better intelligence attributes. Advanced machine learning techniques, which include ANNs [97,98], have received a lot of interest because of their practical uses in important tasks such as image identification and speech processing [99]. NNs employ a lot of multiply–accumulate (MAC) operations, which puts a huge amount of pressure on current electronic computing technology (e.g., CPU, GPU, FPGA, ASIC). For MAC activities, application-specific apparatuses are recommended. Most NNs depend solely on real-valued arithmetic, even though complicated arithmetic might provide a large benefit. For example, a single complex-valued neuron with orthogonal decision boundaries can help resolve the symmetry challenge and the XOR issues, while a single real-valued neuron cannot [100]. Research indicates that complex-valued arithmetic [101] might help NNs function better by providing extensive representational capacity, quick convergence, powerful applicability, and noise-resistant memory mechanisms. Since complex numbers must be formed by two real numbers, which fuels the growth of MAC operations—the most commonly frequently utilized computationally expensive components of NN algorithms—conventional digital electronic computing platforms experience considerable slowdown when implementing algorithms utilizing complex-valued operations [102,103]. To circumvent these difficulties, it has been suggested that the computationally demanding process of building NNs be delegated to OC, which is proficient in genuinely complex-valued arithmetic [74].

Low power consumption, fast processing time, huge data storage, and intrinsic parallelism are all benefits of OC that cannot be matched by its electrical cousin. Numerous optical NN algorithms have been suggested. Photonic chip-based optical NNs, for example, have grown extremely popular due to their remarkable adaptability, scalability, and durability. This system has previously shown neuromorphic photonic weight banks [104], all-optical NNs [105], and optical reservoir computing with tremendous results [106]. On an embedded silicon photonic device, a typical fully connected NN was realized experimentally [107]. Although this optical device is established on light interference, the NN algorithms used are real-valued, negating the merits of sophisticated NNs. The optical impulses were already transformed to photocurrents before entering the accumulator, resulting in a highly parallelized optical NN accelerator established on photoelectric multiplication, which was also built for real arithmetic. On-chip training, optical nonlinear activations [108], and different NN designs are further subjects linked to optical NNs [109].

Apart from OC platforms, analogue electronic apparatuses have effectively exhibited multilayer perceptrons [110,111] and convolutional NNs as compared to more prevalent digital electronic apparatuses [112]. Some earlier research has looked at complex-valued NNs on analogue electrical apparatuses [113,114,115]. Complex-valued reservoirs also result in enhanced system dynamics and enhanced efficiency in reservoir computation. Although optical NNs can handle information in multiple degrees of freedom (e.g., magnitude and phase) using complex-valued numerical methods and acquire more effective information processing and analysis, there have been few investigations in OC platforms for integrating general-purpose and complex-valued NNs [116]. Strongly dependent on traditional deep learning algorithms developed for real-valued arithmetic on traditional electronic computers, existing optical solutions have not walked into this prospective flatland. These real-valued optical NNs are built exclusively on the intensity information of the light waves, ignoring the phase information, which eliminates one of OC’s primary advantages.

**Figure 4:**
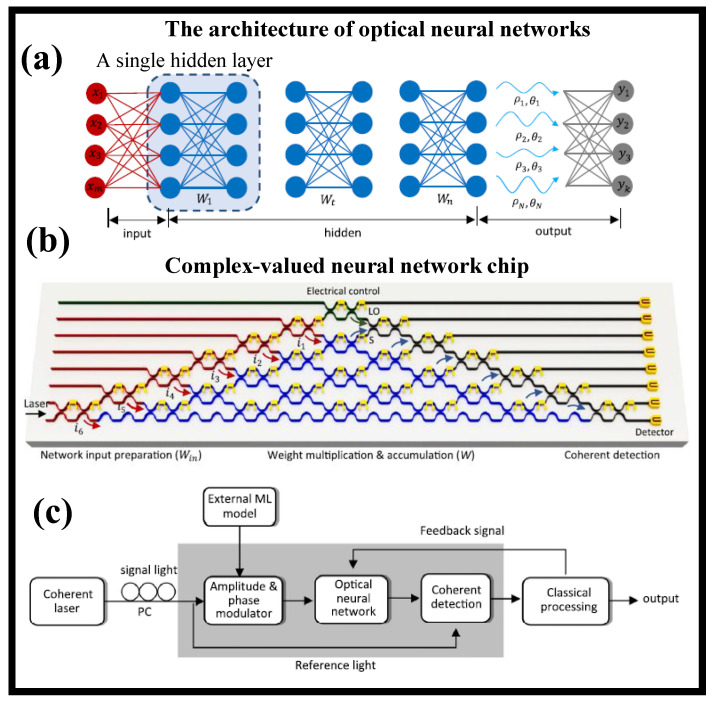
A complex-valued coherent optical NN’s composition. (**a**) An I/P layer, many hidden layers, and an O/P layer make up an optical NN. During the initial I/P preparation and network evolution, the light signals are encoded and modulated by both amplitude and phase in our complex-valued architecture [117]. (**b**) The ONC’s diagram for implementing complex-valued networks. On a single chip, I/P preprocessing, weight multiplication, and coherence recognition are all combined. The MZIs in red are responsible for the division and modulation of the light signals (*i*_1_–*i*_6_). The green MZI distinguishes the reference light that will be utilized for coherent detection later. Blue indicates the MZIs that were utilized to build the 6x6 complex-valued weight matrix. On-chip coherent tracking is established on the remaining gray designated MZIs [117]. (**c**) The ONC system’s process. Signal and reference light is generated using a 1550 nm coherent laser. The amplitude and phase of the signal light on each path are modified by the machine learning (ML) job. The light inference is used to perform the weighted sum process passively. The measurement findings are transferred to the electrical interface for processing, which includes activation function application and cost function computation. The modified weight matrices are then used to reprogram the ONC chip [117].

These problems were answered by designing and demonstrating an optical neural chip (ONC) that performs complex-valued arithmetic, demonstrating the benefits of chip-based complex-valued networks via OC. It has been proved that an optical neural chip (ONC) can build fully complex-valued NNs [117]. The complex-valued ONC’s system is analyzed in four contexts: basic Boolean tasks, species classification of an Iris dataset, nonlinear dataset classification (Circle and Spiral), and handwriting recognition. When referred to its real-valued equivalent, the complex-valued ONC achieves strong skillsets (i.e., precision, quick resolution, and the capacity to generate nonlinear decision boundaries). Figure 4a depicts the optical NN’s architecture, consisting of an I/P layer, many hidden layers, and an O/P layer [117]. During the initial I/P signal preparations and network growth in the complex-valued architecture, light signals are encoded and modulated both by optical magnitude and phase. Figure 4b depicts the ONC design for implementing complex-valued NNs [117]. A single-chip handles I/P preprocessing, weight multiplication, and coherence detection. The I/P signals are generated using a coherent laser (λ = 1550 nm). The ONC is simply a multiport interferometer with a special arrangement of Mach–Zehnder interferometers (MZIs). Each MZI is made up of two-beam splitter–phase shifter (BS–PS) pairs. The BS has a stable transmittance of 50:50, while the PS is thermally regulated to modify the phase. MZIs highlighted with various colors in the figure have distinct capabilities. The bottom point couples the light into the chip. The red chain of MZIs is responsible for I/P light splitting and modulation. The green MZI label distinguishes the baseline light that will be utilized for coherent detection. The on-chip light division ensures that light signals flowing through multiple optical routes are polarized similarly and have a constant relative phase. The machine learning job determines the modulation of the I/P. For jobs involving real-valued I/Ps, the amplitude of the light signals is modulated, and the relative phases between distinct pathways are reduced to 0.

On-chip coherent tracking is based using the gray MZIs. The optical chip’s O/P light signals contain both magnitude and phase information, whereas traditional intensity monitoring systems merely retrieve magnitude data. The intensity and coherent detection are both possible with the integrated chip. The purpose of coherent tracking is to find the phase angles between both the reference and signal light. The O/P current achieved by linking photodiodes at both O/Ps in a balanced manner is *I_1_*α2*A_s_A*_1_cosф*_s_*, where *A_s_* and *A_1_* are the signal and reference light amplitudes, respectively. Likewise, if the baseline light is phase-shifted by π/2, the O/P current is *I_Q_*α2*A_s_A*_1_sinф*_s_*. The ф*_s_* is then calculated using the ratio of *I*_1_ and *I_Q_*, which also aids in the removal of physical noise from optical components. The activation analysis determines which detecting approach is used. A transimpedance amplifier (TIA) converts the recorded photocurrents into voltage signals, which are subsequently gathered and analyzed by a traditional processor with an analog-to-digital converter (DAC). As demonstrated in Figure 4c, feedback signals may be created and routed back to the ONC to alter chip layouts [117].

### 4.4. Diffractive NN

Prior works to OLGs focused primarily on constructive/destructive interference effects between the I/P light signals, encompassing linear [118,119,120,121] and nonlinear interference [122]. The reported works are heavily reliant on accurate positioning of the basic characteristics of two I/P light signals, the control light and/or the pump light, such as phase difference, polarization, and intensity; if the two nanowires are close to each other, as in the plasmonic logic gate, there is also a mandatory rule on the size of I/P beams of light to prevent a big false I/P. Consequently, greater tight control of I/P light may more fully actualize constructive or destructive interference, resulting in a bigger intensity contrast ratio between the two O/P optical logic states “1” and “0”, which is a critical quality to evaluate an OLG’s performance. The heavy dependency on precise I/P light management has two negative effects on the development of miniaturized OLGs. First, the substantial optical components required to perform these controls are considered, and downsizing becomes challenging. Second, because of the complexities of achieving perfect I/P light control, their performance may be unstable, and the intensity contrast ratio between two O/P logic states may become rather low in practical circumstances. It is therefore very desired for compact OLGs to eliminate these important I/P light needs. Due to the necessity of developing innovative designs for all-optical apparatuses and systems, such a goal remains an open problem that has long been sought after.

All seven fundamental optical logic operations (OLOs) are realized in a small system utilizing just plane waves as the I/P signal, thanks to a simple yet universal design method called a diffractive NN [105]. A compound Huygens’ metasurface (MS) implements the diffractive NN, which may somewhat imitate the functions of an ANN [123]. After training, the compound MS can disperse or focus the I/P encoded light in one of two tiny areas/points, one representing logic state ‘1′ and the other representing logic state ‘0′. Three basic OLGs, NOT, OR, and AND, are experimentally confirmed at microwave frequency utilizing a two-layer high-efficiency dielectric MS as a conceptual example. There are two significant advantages to the design technique. First, the implementation of OLOs here eliminates the need for sophisticated and exact control of I/P light characteristics, which sets this technique apart from earlier work. Furthermore, the I/P layer’s architecture is quite broad and strong, and it can be easily changed into numerous user-friendly and programmable formats. Second, if the transmittance state of the I/P layer is dynamically tunable, for example, electrically tunable if the optical mask is generated by a spatial light modulator (SLM) [124], the suggested technique can enable comprehensive logic functionality in a single optical network.

**Figure 5:**
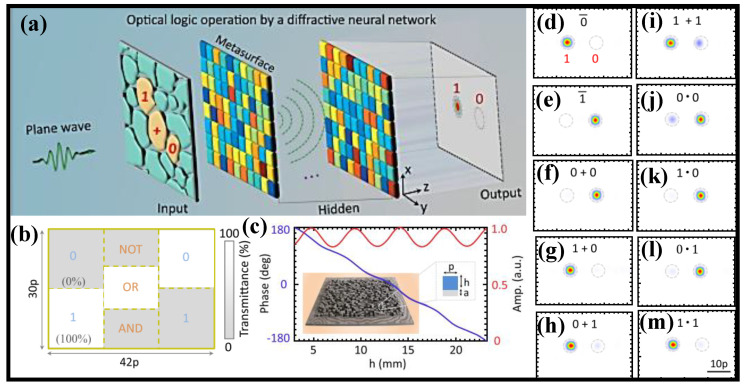
(**a**) A diffractive NN is laid out for photon-based logic functions. A diffractive NN is used to numerically demonstrate three basic logic operations: NOT, OR, and AND. Two levels of MSs make up the hidden layers here [125], (**b**) The I/P layer’s diagram. The white (gray) region’s light transmittance is set to 100% (0%), (**c**) The MS’s transmittance response, which is made up of a 2D array of subwavelength meta-atoms. Each meta-atom may adjust the incoming light’s phase (blue line) and amplitude (red line) locally [125], (**d**–**m**) Intensity distribution for three logic operations with random I/P logic states at the O/P layer. The O/P optical logic state is specified as “1” (“0”) if the field is concentrated on the tiny, prescribed areas on the left (right). Two dashed circles in each panel highlight the selected regions [125].

The I/P layer is a common optical mask that is designed to generate numerous zones, as shown in Figure 5a [125]. Each optical mask zone is set to have two alternative states for optical transmission without sacrificing generality, and its high (low) transmittance state signals whether it is (is not) selected for OC. Then, merely by allocating each of the seven fundamental optical logic operators and the I/P logic states to a specific region, it is simple and efficient to directly specify all seven basic optical logic operators and the I/P logic states in the optical mask. The hidden layers are responsible for decoding the encoded I/P light and rendering the computed result at the O/P layer. The pattern of the I/P layer is shown in Figure 5b. For the sake of simplicity, each region’s high (low) transmittance condition is considered to have a transmittance of 100% (0%) [125]. A cascaded two-layer transmission MS with an axial spacing of 170λ_o_ is used to create the concealed layers (one of the tunable parameters in the training process of diffractive NN). Each MS is made up of 30 × 42 meta-atoms (inset in Figure 5c) [125], each of which has a 0.570λ_o_-width square cross-section. Taking use of its unique qualities such as high transmittance and polarization insensitivity, a simple yet practical high-efficiency dielectric MS is developed. The calculated field intensity after training is depicted in Figure 5d–m [125]. Most of the fields are appropriately concentrated within one of two tiny, specified zones, as intended.

### 4.5. Photonic Crystal (PhC) All-Optical Logic Gates (OLGs)

With the progress of science, there seems to be a significant change in computing in many ways over the years. Mechanical methods were used to create the earliest computers (1623 to 1945) [126]. Researchers attempted to create apparatuses that could readily answer mathematical problems in the early seventeenth century. Several scientists, including Gottfried Leibnitz, Wilhelm Schickhard, and Blaire Pascal, attempted to develop a calculator that could handle addition, subtraction, multiplication, and division. George Schertz and Edward Schertz created a system that could handle 15-digit numbers using a 4-bit difference engine. One of the institutions that employed the mechanical computer for punch card technology for the enumeration was the United States Census Bureau, which was designed by Herman Hollerith of the International Business Machines [126].

A wide range of components, including optical gates, optical switches, optical interconnects, and optical memory, are required to form an optical computer. Because of its applicability in ultrafast information processing [127] and the ways to carry out various logical operations in OC systems, all-optical LGs have become popular recently [128]. As a result, building all-optical LGs is the first step in achieving advanced digital functionality in optical computers. Electronic LGs were previously employed, but the highest switching speed obtained was 50 ps with an average power of 0.5 mW per switching [129]. The reduced capacitance of p-n junctions in semiconductor-based LGs is the explanation for this.

Despite today’s electronic LGs being tiny, switching is still restricted by interlinking capacitance; on the other side, optical LGs have switching speeds in the femtosecond range and are only restricted by the speed of light traveling through them [130]. The prototype of all-optical LGs can be made by a variety of methods. The first technique employs a semiconductor optical amplifier (SOA), which has a high gain owing to refractive index variations. The original way to make all-optical LGs was to use one of three approaches to introduce nonlinearity: cross-gain modulation [131,132], cross-phase modulation [131], or four-wave mixing [132,133]. SOA was also utilized to construct SOA-assisted interferometer-based gates, which are all-optical LGs [134]. However, SOA-based gates have several drawbacks, including SOA-based apparatuses being constrained by SOA’s slow carrier recovery time, unsteady gates owing to polarization sensitivity, and the SOA Mach–Zehnder interferometer (MZI) technique, which necessitates more than two SOAs and complicates the scheme by needing the proper tuning of the filter for SOA with the help of fiber LGs, as detuning of the filter. Nonlinear WGs, in which localized nonlinear media have been used by adjusting the control power, are another way of developing all-optical LGs [135,136,137]. Nonlinear WG-based gates have several drawbacks, including a substantial I/P signal power need and polarization dependency, which pose production issues.

PhCs are periodic structured dielectric or EM media with photonic bandgaps (PBGs) that prevent light from propagating through them [138,139,140,141]. John was the one who introduced PhC [142]. Unlike semiconductor crystals, which alter the characteristics of electrons, these crystals impact the characteristics of photons. Light has various benefits over electrons, including the ability to move faster in dielectric material than electrons in the conductive metal and a larger data capacity in the dielectric than electrons. One of the most significant functions in the realm of high data transmission that 2D PhCs may achieve is all-optical LGs. Furthermore, there are two main types of PhC-based gates: PBG-based gates and non-PBG-based LGs.

(a) Non-PBG-based all-OLGs

**Figure 6:**
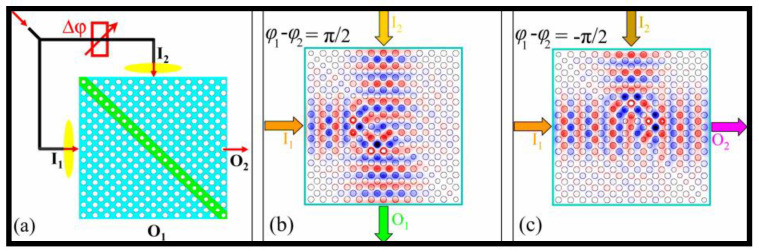
(**a**). The switch’s schematic representation. On the I/P faces I_1_ and I_2_, two beams with different phases collide. The computed steady-state field pattern of the E-polarized mode at 0.194 (a/λ) when incoming beams propagate in the Г-M direction in (**b**,**c**). The phase difference between the two incident beams is adjusted to π/2 and −π/2, respectively, by the phase difference φ_1_ − φ_2_ [143].

Instead of establishing the PBG of the apparatuses, the I/P beam of any wavelength is injected at the I/P, and a logic function is conducted utilizing a self-collimated beam (SCB) in non-PBG-based PhC gates. In this case, incoming light transmits to a device in a given direction without diffraction. The phenomena of total internal reflection are utilized to construct all-optical LGs utilizing an SCB. Total internal reflection refers to the angle of incidence being larger than the critical angle, according to the relation ϴ > arc sin(*n_L_*/*n_H_*), where *n_L_* represents the low refractive index and n_H_ represents the high refractive index. In a 2D PhC, a device for the photonic-integrated circuit (PIC) was formulated and established on an SCB [143]. The structure is useful for making optical switches and LGs, both of which are important parts of a PIC. The device with Si rods in the air was designed using square lattice geometry. To achieve the outphasing at the O/P, a low refractive index medium was created such that one-half of the beam was allowed to propagate while the other was reflected, suggesting the existence of transmitted and reflected beams for the I/P signals. By altering the phase difference between the reflected and transmitted beams, the OR and XOR gate structures were developed. When the phase difference between the I/P beams was 2*k*π + π/2, O/P O_1_ functioned as an OR gate and O/P O_2_ functioned as an XOR gate, as illustrated in Figure 6a–c [144]. O_1_ worked as an XOR gate and O_2_ acted as an OR gate when the phase difference was 2*k*π–π/2 owing to a phase difference of –π/2 between the I/P beams. The device’s frequency range was 0.188 to 0.199, with a 17 dB extinction ratio.

For the PIC implementation, an all-OLG architecture of NOT, OR, AND, and XOR gates centered on the SCB was presented [144]. The structure was constructed using 2D square lattice geometry with air holes in Si as the foundation material. The framework has been developed and established on the phase difference between the I/P signals, following the phenomena of the SCB. When light is launched at both the I/P as well as reference points of the same intensities, there was partial reflectance of the I/P light wave while the reference light wave was completely mirrored, and when these light waves interfered with each other, there was an O/P that relied on the phases at the I/Ps and interfered in a constructive manner or destructively. It was proposed to use SCB-based logic gates for AND, NAND, XNOR, and NOR OLGs. The framework has been developed with triangular lattice topology and rods made of Si material in an air background [145]. Table 1 shows that different studies offered alternative architectures for creating various gates. Most OLGs were created in [144], which implemented AND, NAND, XNOR, and XOR OLGs. Si rods in an air environment are the structure utilized by all researchers. However, in the case of CR, the structure developed in [145] for NOT gate produced the greatest results, i.e., 30 dB. Furthermore, OLGs constructed with a self-collimated light wave have a few drawbacks, including a low contrast ratio (CR), a wide area, a high cost owing to the big size, and signal guiding in only the vertical and horizontal directions.

(b) PBG-based all-OLGs

**Figure 7:**
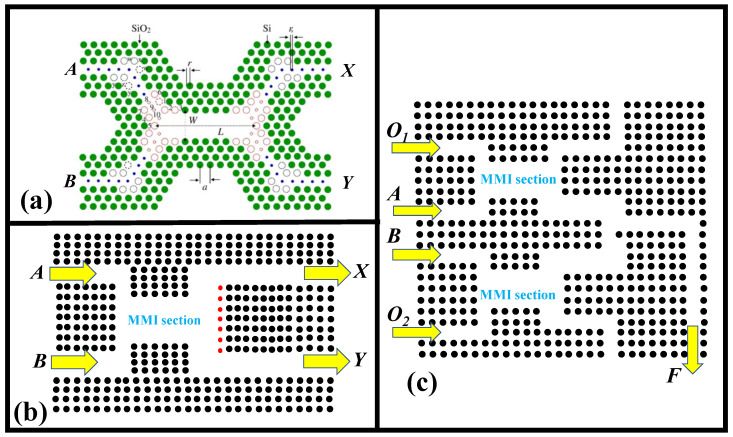
Schematic of OLGs established on MMI. Inspired by [146], (**a**) AND/XOR OLG, (**b**) XOR/XNOR, (**c**) AND and NOR OLGs. Inspired by [150].

The PBG of the architecture is utilized to detect hidden frequency ranges that cannot flow through the structure in PBG-based all-optical OLGs. By adding various forms of defects, one of the suppressed frequencies can transmit across the structure. MMI, nonlinear Kerr effect, and interference are commonly used in the design of PBG-based OLGs. In [149], MMI-based AND and XOR OLGs are introduced, as depicted in Figure 7a, with A and B I/P points and X and Y O/P points. It was constructed utilizing triangular lattice geometry, SiO_2_ as the foundation material, and Si rods. An I/P signal with an outphasing of π was transmitted at point A. On point B, the signal was emitted with an outphasing of –π/2, which represented logic “1” and creates logic “1” at the O/P point. When there was a phase of π at point *A*, which indicated logic “0”, and another signal with outphasing π/2 at point *B*, which expressed logic “0”, the O/P generated logic “1”. In another scenario, when a signal with an outphasing of π expressed logic “0” at I/P point *A* while a signal with an outphasing of π/2 stated logic “0” at point *B*, logic “0” was identified at the O/P. When both I/Ps at point *A* and point *B* displayed logic “1” with outphasings of 0 and −π/2, the O/P point sensed logic “0”. The AND OLG was constructed using the same concept by modifying the length of MMI and appropriately choosing I/P signal phases. Both AND and XOR OLGs obtained a CR of roughly 6.79 dB.

The self-imaging mechanism underpins the operation of MMI apparatuses. The tiny field at the I/P activates guided modes in the effective region, which constitutes interference in that region, in this phenomenon. Binary phase-shift keyed (BPSK) signals are utilized as I/P logic values because, in MMI, I/P values are often expressed by the phase of the I/P signals, and O/P logic levels are expressed using amplitude independent of phase. Silicon material was employed for the rods in the suggested device, with air as the background oriented in a square lattice shape [150]. As indicated in Figure 7b, A and B are the I/P points of the XOR/XNOR OLG arrangement, whereas X and Y are the O/P points. When there is an outphasing of π at point A, logic “0” was stated as signal I/P, while logic “1” is explicit with phase 0. When there was an outphasing of 3π/2 at point B, logic “0” was expressed as signal I/P, while logic “1” was explicit with phase 0. To realize the AND OLG indicated in Figure 7c, I/Ps A and B with phase π represented logic “0,” whereas I/Ps A and B with phase 0 represented logic “1”. With an outphasing of 3π/2, logic “0” was established at O_1_ and O_2_. The sole change in designing the NOR OLG was that O_1_ and O_2_ were locked at logic “1”, which was represented by an outphasing of π/2. The AND OLG realized a CR of 21 dB, whereas the NOR operation achieved a CR of 19 dB [150]. Table 2 demonstrates the effectiveness of PBG-based all-OLGs on performance metrics such as the CR. PBG-based OLGs established on the interference phenomenon have a higher CR and are easier to construct than OLGs established on other concepts [151,152,153,154,155,156,157,158].

### 4.6. Resonant Nanophotonic Constructions

Nanophotonic components are being suggested as a novel foundation for analog optoelectronic computing [159,160,161,162]. It was shown that a layer of a well-designed metamaterial can visually execute several essential mathematical operations (differentiation and integration of light waves concerning a spatial coordinate, convolution of light waves with a predefined core) [160]. It generated a lot of interest and aided the creation of novel nanophotonic structures for AOC. Differentiation (integration) of the pulse envelope is commonly understood in the context of temporal differentiation (integration) of a light wave (optical pulse). The most important findings in fiber optic network design and production for the differentiation (integration) of optical pulses propagating in OFs were acquired [163,164,165,166,167,168,169]. Bragg resonant structures and RRs have been proposed to perform time-domain differentiation (integration) procedures. Because the spectra of reflection and transmission in the proximity of resonances are characterized by the Fano profile, and in a certain frequency, intervals may accurately estimate the transfer functions of differentiating and integrating filters, resonant formations can be used to implement differentiation and integration operations. It is worth noting that more complex systems with many resonators may solve ordinary differential equations of various orders as well as solutions of differential equations in the temporal domain [170,171,172,173].

The employment of differentiation and integration of light pulses traveling in free space is also of importance, in addition to the temporal changes of light signals traveling through OFs. Soifer et al. achieved several significant discoveries in this field. The processes of differentiation (integration) of the pulse envelope may be successfully conducted by employing a resonant diffraction grating, as described in studies [174,175,176,177,178,179]. High-order derivatives may be calculated quickly using a set of multiple stacked diffraction gratings [178]. The creation of the theory of spatiotemporal transformations of light waves was established on the theoretical explanation of diffraction of light pulses on resonant diffractive assemblies.

An optical correlator can conduct a wide range of spatial filtering functions on light waves. The optical correlator, also known as the coherent optical Fourier processor, is made up of two lenses that execute the FT optically and a spatial filter that encapsulates the transmission function characterizing the incoming light beam’s needed spatial transformation. As a spatial filter, a differentiating filter with a complicated transmission function corresponding to the spatial frequency should be utilized to accomplish the differentiation operation in such a scheme. Nevertheless, the optical correlator’s significantly larger size limits its practical applicability. Using nanophotonic configurations, spatial differentiators and integrators with a thickness similar to the wavelength of the modulated light wave may be created. In 2014, the idea of implementing spatial changes of light waves utilizing nanophotonic apparatuses was introduced. The fundamental work in [159] was a theoretical investigation of the execution of spatial differentiation, integration, and convolution operations on light waves. There were two ways offered. The first was to employ tiny analogues of optical correlators, with the traditional Fourier lenses being substituted by tiny layers with a gradient refractive index and an MS serving as the spatial filter storing the requisite transmission function. The second method was to utilize a multilayer structure that was particularly built to accomplish the spatial transformation of the I/P signal indicated by the convolution operator with a certain core.

Bozhevolnyi et al. conducted the first study proving the capability of differentiation and integration regarding a spatial factor using an MS in 2015 [162]. The first strategy was utilized for optical differentiation and integration, in which the processes were carried out in an optical correlator comprised of a lens and a reflecting spatial filter. A reflecting MS encrypting transmission function of a differentiating (integrating) filter was employed as the filter. The MS in question was made up of a series of the metal–insulator–metal resonant circuit [16,180]. Fabry–Perot resonances of plasmonic modes traveling in metal slots are supported by such resonators. The experimental findings provided in [162] show that optical differentiation (integration) may be implemented with the use of an MS. Simultaneously, it should be highlighted that the MSs in [162] only conduct differentiation and integration tasks in conjunction with a lens, preventing the suggested system from being compact. In subsequent work [181,182,183], dielectric MSs were used to encode the essential transmission functions of the spatial filter, causing a reduction in losses due to absorption in the metal claddings of nano-resonators and enhanced performance in the deployment of differential and integral conversions.

Because the nanophotonic assembly directly executes the needed spatial change of the I/P signal, the second option is more plausible. In this example, the optical correlator is replaced with a single assembly (with no extra lenses). It is worth noting that the structure’s reflection and transmission coefficients as functions of spatial frequency (a tangential component of the incoming wave vector) match the transfer functions defining the incident light beam’s conversion [162]. As a result, nanophotonic assemblies with reflection (or transmission) coefficients that approximate the transfer functions of the differentiator or integrator would be required to accomplish the elementary actions of spatial differentiation or integration of the light beam. A resonant reflection or transmission band with a Lorentzian line form can be used to mimic the integrator’s transfer function. About the zeros of reflection and transmission happening near the resonance, the differentiator’s transfer function is very well modeled.

Soifer et al. achieved the most important results in this sector. The work of this group initially established that phase-shifted Bragg gratings (PSBGs) [184,185,186], resonant diffraction gratings [187,188], PhC resonators [189,190,191,192,193,194], and three-layer arrangements with W-shaped refractive index profiles may efficiently execute the functions of differentiation and integration of the light wave profile [195]. It is worth noting that resonant nanostructures can also be used to compute the Laplace operator optically [186]. In image processing, this method is utilized to recognize edges. The first investigation affirming the potential of differentiating a spatial variable using the resonant diffraction grating was carried out in 2018 using the apparatuses of the Collective Use Center “Nano-Photonics and Diffraction Optics” [196], which was established by a cooperative endeavor of Samara University and the Russian Academy of Sciences’ Image Processing Systems Institute [195]. It resulted in a high level of distinction that was far superior to the quality of differentiation attained using MSs [162]. It is also important to note that differentiators and integrators founded on resonant diffraction gratings and PSBGs are not only smaller but also easier to fabricate than equivalent apparatuses that rely on correlators with MSs. The emphasis of [197] is a differentiator comprising of a prism with a metal film placed on one of its sides; specifically, the differentiator is composed of a prism with a metal film coated on one of its sides [198,199]. The incident light wave’s differentiation is carried out in this scenario in reflection due to the incident light wave’s activation of a surface plasmon-polariton on the metal film’s surface. The effectiveness of employing such a framework for optical edge detection was proved in experiments.

**Figure 8:**
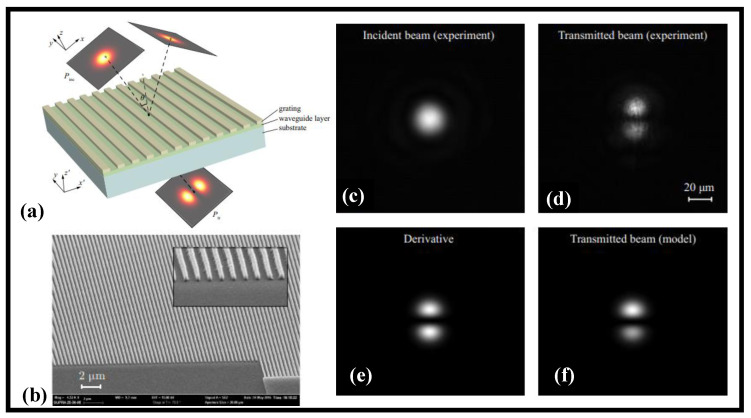
(**a**) The diffraction of an optical light wave on a resonant diffraction assembly made up of a grating on top of a slab waveguide layer put on a substrate [188], (**b**) The diffraction grating was created using ERP-40 electron resist on top of a TiO_2_ layer, as shown by SEM [188], (**c**) Measured profile of incident Gaussian light wave [188], (**d**) Measured profile of transmitted light wave [188], (**e**) Analytically derived derivative of incident light wave [188], (**f**) Profile of transmitted light wave estimated taking into account manufactured structural flaws [188].

The first-order differentiation of the transverse profile of an incoming light wave regarding a spatial variable is demonstrated experimentally using a subwavelength diffraction grating, as shown in Figure 8a [188]. Figure 8b shows a typical SEM picture of the manufactured grating. The experimental findings accord well with the provided theoretical model, implying that the differentiation occurs in transmission at oblique incidence and is linked to the grating’s guided-mode resonance. As per this concept, the grating’s transfer function about the resonance is similar to that of an exact differentiator. Figure 8c,d illustrates the incident Gaussian light wave and transmitted light wave profiles, respectively. The incident light wave has a Gaussian shape to it. The precise derivative agrees well with the form of the transmitted light wave (Figure 8e). The profile of the transmitted light wave estimated considering manufactured structural flaws is also shown in Figure 8f. The configuration under consideration might be used in the development of novel photonic apparatuses for light wave shaping, optical data processing, and AOC.

## 5. Growth Ideas, Constraints and Misconceptions

For the many methods of OC, there are certain common obstacles. To begin with, the large-scale integration of optical–electrical chips must be completed to boost the parallelism of OC systems at the physical level. In addition, optical–electrical co-package technology is required to decrease the price of data transport between the electrical and optical domains. Second, current optical transmitters and modulators are primarily intended for optical transmission rather than processing. Because most implementations demand high bit depth I/P data, OC methods allow a substantially greater extinction ratio (ER) and linearity of optical apparatuses than optical communication. Furthermore, because optical apparatuses with a greater ER and linearity may allow high-efficiency optical coding for data I/P, the overall performance will be enhanced. Eventually, a new architectural design is necessary. The optical–electrical transformation might severely limit the energy efficiency of the hybrid computing device, making it impossible to make use of the benefits of OC in a traditional computer design. The new architectural design might have a significant speed-up factor while retaining as much customizability as feasible in the meantime. Finally, there are a few investigations on algorithms that are appropriate for analog OC. Algorithms are now built using Boolean logics, which are ideal for digital computer systems. They are, nonetheless, impossible to match when it comes to the functionality given by OC. If algorithms for OC are created, their operation overhead and execution time will be significantly reduced compared to present methods.

Despite the numerous hurdles, OC’s potential has been growing. To begin with, various manufacturing methods have been involved in the development of larger-scale optical–electrical chip integration. For instance, Light-matter delivered the world’s first 4096 MZI integrated chip, called ‘Mars,’ demonstrating the capability of large-scale integration and giving researchers in OC more confidence. Furthermore, the previously described WDM and MDM, as well as the spatial optical system, are all sustainable with increasing parallelism. Moreover, by directly employing a faster-speed optical device with low-bit depth optical coding, the poor ER and linearity of optical apparatuses may be adjusted. In terms of data I/P performance, a 2 GHz optical modulator with OOK and a 1 GHz optical modulator with PAM4 are equal. This type of compensation, on the other hand, is only possible in computer processes that can be transformed into a linear combination of low-bit depth operations in the time domain. Using low-bit depth quantization for application I/P data, on the other hand, is a common method for making current optical instruments practical in OC.

Lightwave looping should be extensively used to maintain data in the optical domain for as prolonged as feasible in hybrid computing systems to decrease the overhead from optical–electrical conversion. The temporal delay induced by light wave looping might be minor due to the fast propagation speed of light. New designs can be inspired by dataflow approaches. Finally, OC techniques might consider the complicated operators available in the optical domain. To minimize implementation risk and processing time, several sets of Boolean logic operators in present algorithms can be substituted with a single complicated operator. As a result, integrating complicated operators with Boolean logic operators in an algorithm might be a promising technique to construct OC algorithms. OC’s possibilities have been expanding. The ever-increasing need for ANNs, as well as their processing requirements, will continue to push research into OC patterns. Optical sensing and communication may provide another opportunity for OC to be used. Furthermore, high-complexity computing algorithms in the optical domain, including FT, convolution, and equation solving, might significantly improve systematic efficiency. Some of the important applications of OC and current state-of-the-art research works are summarized in Table 3.

Investigators from the universities of Oxford, Exeter, and Münster have developed a novel technology that allows them to retain more optical data in a smaller space on-chip than formerly conceivable. This method achieves the phase-change optical memory cell, which utilizes light to record and read data, and it might result in a quicker, more energy-efficient computer memory. Unlike today’s computers, which utilize electrical impulses to store data in one of two states-zero or one-the optical memory cell stores data via light. The researchers achieved optical memory with over 32 states, or levels, equating to 5 bits. This is a significant step toward the development of an all-optical computer, which is a long-term aim for many researchers in this field [200].

In the coming years, we can expect the creation of all-optical computers in the form of research samples; however, industrial prototypes that have a market niche require the implementation of effective technological solutions in electronics. Nevertheless, the advantages of photonic solutions over electronic ones listed in the review paper make it necessary for scientists to continue research in this direction and systematize the accumulated results. From the point of view of the authors, at present, solutions in the optical implementation of certain types of special processors may turn out to be effective, for example, replacing the used electronic analog machines with similar optical ones providing the solution of certain types of differential equations, contour detection, etc. The proposed solutions can be promising where initially there is an optical signal at the input of a special processor, for example, a video stream. In this case, the optical neural network allows processing and recognizing some images faster than converting them into electronic form and processing it on a digital computer using graphics accelerators.

The fact that processing is a nonlinear process in which several signals must interplay is a key problem for optical computing. Light, which is an electromagnetic wave, can only engage with another electromagnetic wave in the possession of electrons in a material, and the intensity of this interaction is significantly less for electromagnetic waves than for electrical signals in a traditional computer. As a response, processing elements for an optical computer may require more power and have a bigger size than processing elements for a traditional electronic computer employing transistors. Another myth is that optical transistors should be susceptible to incredibly high frequencies, since light travels far faster than electrons’ drift velocity and at frequencies measured in THz. The pace at which an optical transistor may react to a signal is still restricted by its spectral bandwidth, since every electromagnetic wave must respect the transform limit. Practical constraints such as dispersion frequently restrict fiber-optic communications channels to bandwidths of tens of GHz, which is just marginally better than many silicon transistors. To achieve far quicker operation than electronic transistors, effective means of sending ultrashort pulses along extremely dispersive waveguides would be required.

## 6. Conclusions

Artificial neural networks (ANNs) have been used to build artificial intelligence in recent times, resulting in unimaginable demands for computing resources. Nevertheless, due to the insufficiency of Moore’s law’s and the failure of Dennard’s scaling laws, traditional computer hardware related to electronic transistors and von Neumann architecture would be unable to meet such an incomprehensible demand. Conversely, analog optical computing (AOC) provides an alternate method for unleashing enormous processing power to speed up a wide range of compute-intensive operations. To perform specific computing operations, AOC makes use of physical features of light, including amplitude and phase, as well as the interplay between light and optical devices. Because of the unique mathematical portrayal of computational processes in one specific analog optical computing system, it is a specialized computing system. AOC can achieve higher data processing acceleration in specialized applications, including pattern recognition and numerical calculation, as opposed to traditional digital computing. The popularity of optically implemented neural networks has grown in recent years, which is attributable to the growing quantity of databases that must be handled, placing a strain on the effectiveness of traditional digital, electronic computers. The key consideration in implementing a viable optical computer (including a neural one) is to integrate the linear part of the system, from which optics derives its competitive advantage, with nonlinear components and I/P–O/P interfaces while preserving the optical interconnections’ speed and power efficiency. In this paper, several optical components such as spatial light modulators, plasmonic switches, neural networks, diffractive neural networks, photonic crystal all-optical logic gates, and resonant nanophotonic structures that are used to implement optical computing have been reviewed, and their advancements have been discussed. We believe that this paper will be beneficial to the scientific community working on the topic of optical computing.

## Figures and Tables

**Table 1:** Evaluation of different kinds of non-PBG-based all-OLGs.

Logic Operation	Operational Wavelength (nm)	Lattice Type	Polarization	CR (dB)	Ref.
OR, XOR	-	Square (rods in air)	TE	17	[143]
WG for OLGs	1550	Square (air holes in Si)	TE/TM	-	[146]
NOT, OR, AND, XOR	1550	Square (rods in Si)	TE	30	[144]
AND, NAND, XNOR, NOR	1555.1	Square (rods in air)	TE	6	[145]
XOR, OR	1550	Trianular (rods in air)	-	-	[147]
AND	1550	Triangular (rods in air)	-	-	[148]

**Table 2:** Device performance of different types of PBG-based all-OLGs.

Logic Operation	Operational Wavelength (nm)	Lattice Type	Polarization	CR (dB)	Ref.
XNOR, XOR, OR, NAND	1550	Square (rods in air)	TE	37.4–40.41	[19]
NOT, AND, OR, XOR, XNOR, NAND	1550	Triangular (holes in Si)	TE	NOT-3.74, AND-11.47, OR-12.48, XOR-6.50, XNOR-6.50	[47]
XOR, XNOR, NAND, OR	1530–1565	Triangular (rods in SiO_2_)	TM	XOR-28.6, XNOR-28.6, NAND-25, OR-26.6	[151]
NAND	1554	Square (rods in air)	TM	-	[153]
OR, AND	OR-1529, AND-1538	Triangular (rods in air)	TM	6	[155]
NAND, NOR, XNOR	1550	Square (rods in air)	TE	17.59, 14.3, 10.52	[156]
NOR, AND	1550	Triangular (rods in air)	TE	-	[157]
OR	1287.8	Triangular (rods in air)	TE	7.27	[158]

**Table 3:** Some important applications of OC.

	Applications	References
1	Parallel processing	[201,202,203,204]
2	Optical switches	[4,205,206]
3	Optical data storage	[200,207,208]
4	Data communication	[209,210]
5	All-optical logic operation	[127,211,212]

## Data Availability

Not applicable.

## Abbreviations

OC = Optical computing; NN = Neural network; OLG = Optical logic gate; PhC = Photonic crystal; DOC = Digital optical computing; AOC = Analog optical computing; ANN = Artificial neural network; MS = Metasurface; ER = Extinction ratio; CR = Contrast ratio; SCB = Self collimated beam; I/P = Input; O/P = Output; BS-PS = Beam splitter–phase shifter; MZI = Mach–Zehnder Interferometer; WDM = Wavelength division multiplexing; MDM = Mode division multiplexing; SOA = Semiconductor optical amplifier.
